# Carnosic Acid Inhibits Herpes Simplex Virus Replication by Suppressing Cellular ATP Synthesis

**DOI:** 10.3390/ijms25094983

**Published:** 2024-05-03

**Authors:** Georgina Horváth, Edit Molnár, Zoltán Szabó, Gábor Kecskeméti, László Juhász, Szabolcs Péter Tallósy, József Nyári, Anita Bogdanov, Ferenc Somogyvári, Valéria Endrész, Katalin Burián, Dezső P. Virok

**Affiliations:** 1 Department of Medical Microbiology, Albert Szent-Györgyi Health Center and Albert Szent-Györgyi Medical School, University of Szeged, Semmelweis Str. 6, 6725 Szeged, Hungary; 2 Réthy Pál County Hospital, Gyulai Str. 18, 5600 Bekescsaba, Hungary; 3 Department of Medical Chemistry, Albert Szent-Györgyi Health Center and Albert Szent-Györgyi Medical School, University of Szeged, Dóm Sq. 8, 6720 Szeged, Hungary; 4 Institute of Surgical Research, Albert Szent-Györgyi Health Center and Albert Szent-Györgyi Medical School, University of Szeged, Szőkefalvi-Nagy Béla Str. 6, 6720 Szeged, Hungary

**Keywords:** rosemary, carnosic acid, herpes, herpes simplex, antiviral, antimicrobial, mitochondrion, oxidative phosphorylation, glycolysis, citrate cycle

## Abstract

Acquiring resistance against antiviral drugs is a significant problem in antimicrobial therapy. In order to identify novel antiviral compounds, the antiviral activity of eight plants indigenous to the southern region of Hungary against herpes simplex virus-2 (HSV-2) was investigated. The plant extracts and the plant compound carnosic acid were tested for their effectiveness on both the extracellular and intracellular forms of HSV-2 on Vero and HeLa cells. HSV-2 replication was measured by a direct quantitative PCR (qPCR). Among the tested plant extracts, *Salvia rosmarinus* (*S. rosmarinus*) exhibited a 90.46% reduction in HSV-2 replication at the 0.47 μg/mL concentration. Carnosic acid, a major antimicrobial compound found in rosemary, also demonstrated a significant dose-dependent inhibition of both extracellular and intracellular forms of HSV-2. The 90% inhibitory concentration (IC_90_) of carnosic acid was between 25 and 6.25 μg/mL. Proteomics and high-resolution respirometry showed that carnosic acid suppressed key ATP synthesis pathways such as glycolysis, citrate cycle, and oxidative phosphorylation. Inhibition of oxidative phosphorylation also suppressed HSV-2 replication up to 39.94-fold. These results indicate that the antiviral action of carnosic acid includes the inhibition of ATP generation by suppressing key energy production pathways. Carnosic acid holds promise as a potential novel antiviral agent against HSV-2.

## 1. Introduction

HSV-2, a member of the *Herpesviridae* family, is known for establishing latent infections in the sacral and trigeminal ganglia post-acquisition. Periodic reactivation of the virus leads to the development of genital ulcers, making HSV-2 a common cause of infections among both immunocompetent and immunocompromised individuals. The World Health Organization reports that approximately 431 million people aged 15–49 are infected with HSV-2 [1]. Active HSV-2 infection can lead to severe complications such as meningitis, encephalitis, and an increased risk of acquisition of HIV infection [2]. While the eradication of HSV-2 is not achievable, the antiviral treatment of acute/recurrent infections is possible with primarily acyclovir and its derivatives. However, acyclovir-resistant isolates emerge especially in immunocompromised patients such as HIV-positive individuals and organ transplant and bone marrow recipients [3]. Therefore, the search for novel anti-herpesvirus drugs is an important task. An ideal antiviral drug or drug combination should inhibit the extracellular virions and intracellular virus replication. According to WHO, “the medical plant is any plant which in one or more of its organs contain substances that can be used for therapeutic purposes or which are precursors for chemimedical semisynthesis” [4]. Medical plants represent a chemical library with a high molecular diversity and structural complexity providing a rich source of antiviral drug research. Plant extracts are an abundant source of metabolites. METLIN, a mass spectrometry database, contained 62,000 spectra describing more than 12,000 plant metabolites [5]. NAPROC-13, a nuclear magnetic resonance (NMR) database contains spectra of 6000 natural compounds including plant metabolites [6]. Therefore, we tested eight plants often used in local medicine in the Southern region of Hungary for their antiviral activity against HSV-2. The following plants were tested: *Melissa officinalis* (*M. officinalis*), *Urtica dioica* (*U. dioica*), *S. rosmarinus*, *Althaea officinalis* (*A. officinalis*), *Sambucus nigra* (*S. nigra*), *Nepeta cataria* (*N. cataria*), *Lavandula angustifolia* (*L. angustifolia*), *Origanum vulgare* (*O. vulgare*). All of the tested plants were shown to have antimicrobial activity against either/or bacteria, fungi, and viruses. *M. officinalis* had antiviral activity against HSV-1, HIV, and SARS-CoV-2 [7]. *U. dioica* showed antiviral activity against feline immunodeficiency virus and rabies virus [8,9]. *S. nigra* significantly inhibited HSV-1 and HIV replication in vitro [10]. *O. vulgare* showed antiviral activity against murine norovirus [11], SARS-CoV-2 [12] and HIV-1 [13]. *N. catania* relatives *N. nepetella*, *N. coerulea*, and *N. tuberosa* were active against HSV-1 and vesicular stomatitis virus [14]. *L. angustifolia* was active against avian influenza H5N1 virus [15]. Our results showed, that among the tested plant extracts, *S. rosmarinus*, commonly known as rosemary, has demonstrated a significant antiviral activity against HSV-2. The antibacterial activity of *S. rosmarinus* extracts is well described [16,17], but there are less data on its antiviral activity [18,19,20].

Carnosic acid, a natural phenolic diterpene found in *S. rosmarinus*, exhibits bioactive properties that impact various infection-related biological processes such as inflammation and apoptosis [21,22]. Studies have demonstrated the antimicrobial effects of carnosic acid against respiratory syncytial virus [23] and influenza virus [24]. In this study, we investigated the antiviral properties of carnosic acid on both extracellular and intracellular forms of HSV-2, revealing significant inhibition of viral replication. Through liquid chromatography with tandem mass spectrometry (LC-MS/MS) proteomics analysis of carnosic acid-treated HeLa cells, we also searched for potential antiviral mechanisms of the compound.

## 2. Results

### 2.1. Impact of Plant Extracts on the Viability of Vero Cells

**Figure 1:**
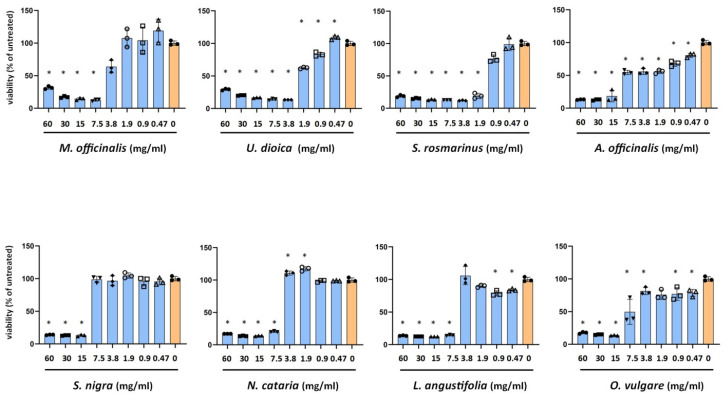
MTT cell viability assay of Vero cells incubated with the plant extracts. Cell culture media of HSV-2 infected Vero cells were replaced with plant extracts resulting in the concentration range of 60–0.47 mg/mL. Vero cells were incubated for 24 h, 37 °C, 5%CO_2_. Viability of the treated cells was compared to the untreated controls. Data are mean ± SD (*n* = 3). Statistical comparisons of cell viabilities (treated vs. untreated control) were performed by one-way ANOVA analysis corrected by Dunnett’s multiple comparison test. *: *p* < 0.05.

In order to assess the cytotoxicity of the plant extracts we incubated Vero cells with a serial 1:2 dilutions of extracts (Figure 1). A toxicity threshold of 75% viability of the untreated Vero cells was applied. The least toxic was the *S. nigra* extract with a 7.5 mg/mL non-toxic concentration. The plant extract showing the highest toxicity was *A. officinalis* with 0.94 mg/mL non-toxic concentration. Thus, the range of non-toxic concentrations was between 7.5 and 0.47 mg/mL. Non-toxic concentrations of each plant were used in further antiviral tests.

### 2.2. Antiviral Activity of Plant Extracts against HSV-2

**Figure 2:**
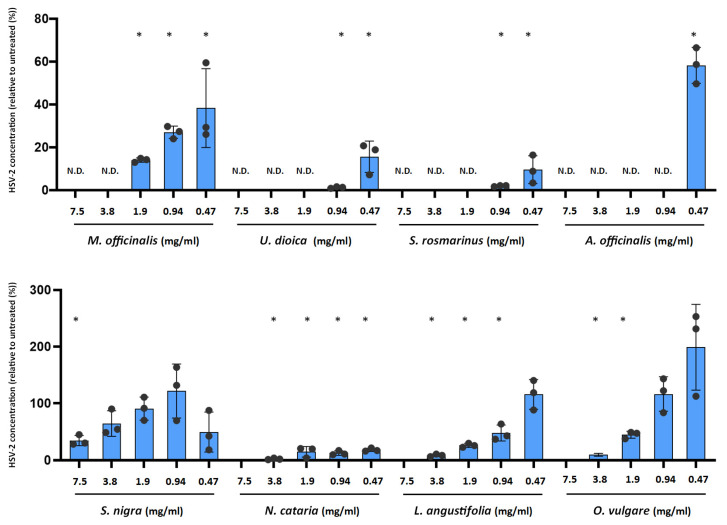
Impact of plant extracts on HSV-2 replication. Cell culture media of HSV-2 infected Vero cells were replaced with medium containing 7.5–0.47 mg/mL of plant extracts. Vero cells were incubated for 24 h, 37 °C, 5%CO_2_. At 24 h post-infection, Vero cells were lysed and a direct qPCR was applied to measure the HSV-2 genome concentrations. Data are mean ± SD (*n* = 3). Statistical comparisons of qPCR Ct values (treated vs. untreated control) were performed by one-way ANOVA analysis corrected by Dunnett’s multiple comparison test. *: *p* < 0.05. N.D: not done.

We previously developed a direct qPCR method to evaluate the impact of antiviral compounds on HSV-2 replication [25]. This method measures the HSV-2 genome concentration as a proxy of viral replication. We infected HeLa cells with HSV-2 and replaced the media with plant extracts in the range of 7.5–0.47 mg/mL. HSV-2 genome concentrations were measured at 24 h post-infection (Figure 2). All the tested plant extracts inhibited the viral replication to a certain degree. At the lowest 0.47 mg/mL concentration, five of the eight tested plant extracts showed inhibition. The highest inhibition was observed for *S. rosmarinus* (90.46 ± 6.52% reduction), *U. dioica* (84.42 ± 7.33% reduction), and *N. cataria* (82 ± 2.93% reduction). The higher 0.94 mg/mL concentration resulted in a close to 100% inhibition of HSV-2 replication in the case of *U. dioica* (98.77 ± 0.39% reduction) and *S. rosmarinus* (98.06 ± 0.24% reduction). *M. officinalis* and *S. nigra* extracts showed significant inhibitory activity only at higher concentrations.

### 2.3. Antiviral Activity of Carnosic Acid against HSV-2 in Vero and HeLa Cells

**Figure 3:**
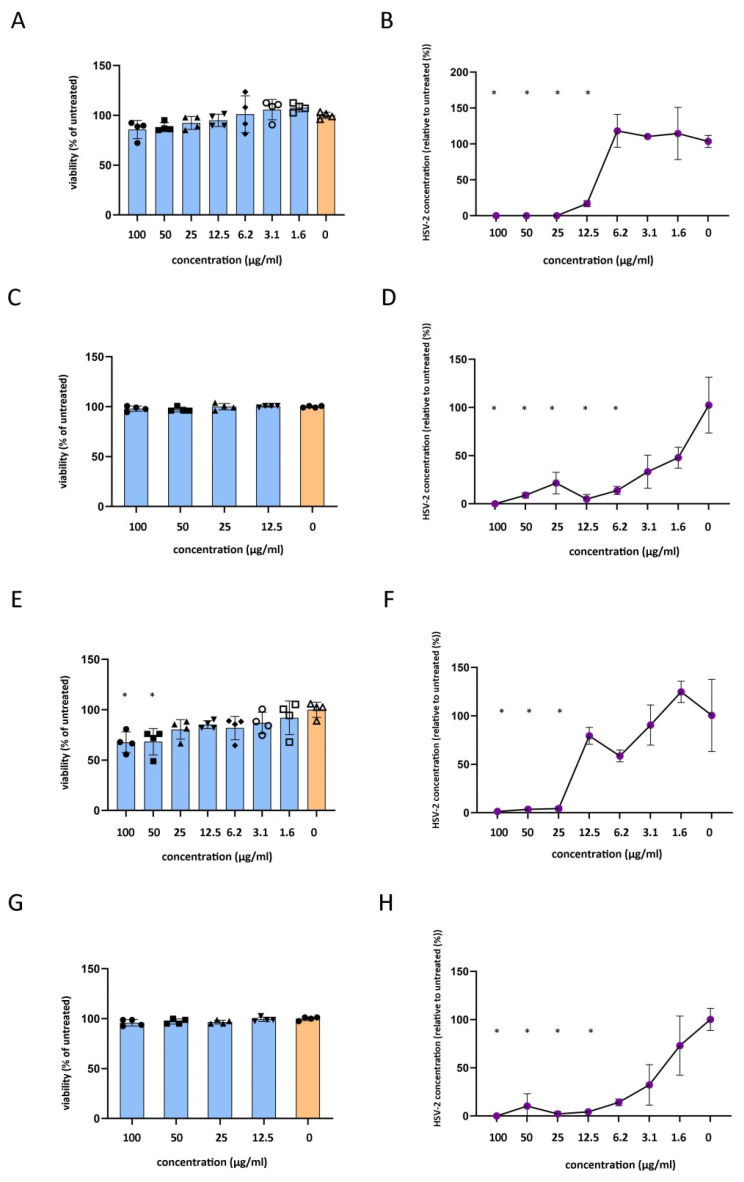
Impact of carnosic acid on host cell viability and HSV-2 replication. (**A**), Impact of 24 h carnosic acid treatment on Vero cell viability measured by MTT assay. (**B**), Impact of 24 h carnosic acid treatment on HSV-2 replication in Vero cells measured by direct qPCR. (**C**), Impact of 30 min carnosic acid treatment on Vero cell viability measured by MTT assay. (**D**), Impact of 30 min carnosic acid treatment of HSV-2 virions on HSV-2 replication measured by direct qPCR 24 h post-infection. (**E**), Impact of 24 h carnosic acid treatment on HeLa cell viability measured by MTT assay. (**F**), Impact of 24 h carnosic acid treatment on HSV-2 replication in HeLa cells measured by direct qPCR. (**G**), Impact of 30 min carnosic acid treatment on HeLa cell viability measured by MTT assay. (**H**), Impact of 30 min carnosic acid treatment of HSV-2 virions on HSV-2 replication measured by direct qPCR 24 h post-infection. MTT data are mean ± SD (*n* = 4), and qPCR data are mean ± SD (*n* = 3). Statistical comparisons of treated vs. untreated samples were performed by one-way ANOVA analysis corrected by Dunnett’s multiple comparison test. *: *p* < 0.05.

To measure the long-term cytotoxicity of the carnosic acid, we incubated Vero cells with the compound in the range of 100–1.6 μg/mL (Figure 3A) for 24 h. None of the tested concentrations induced significant changes in the MTT values, but the 100 μg/mL and 50 μg/mL carnosic acid concentrations induced an observable cytotoxicity by light microscopy. To assess the carnosic acid activity on the intracellular HSV-2, the carnosic acid was added to the cell culture media after HSV-2 infection at a final concentration of 100–1.6 μg/mL for 24 h. qPCR data showed that HSV-2 genome accumulation was significantly inhibited by carnosic acid at the 100–12.5 μg/mL concentrations (Figure 3B). The 12.5 μg/mL carnosic acid concentration resulted in an average of 4.95-fold reduction in HSV-2 concentration, thus the carnosic acid inhibitory concentration 50 (IC_50_) was between the 12.5 and 6.25 μg/mL concentration. The 25 μg/mL carnosic acid resulted in a 1080.5-fold reduction in HSV-2 concentration. Therefore, the carnosic acid IC_90_ concentration was between 25 and 12.5 μg/mL. We also measured the direct inhibitory activity of carnosic acid on HSV-2 virions by preincubating the virions with carnosic acid (100–1.6 μg/mL final concentration). To model this type of cytotoxicity, a 30 min incubation of the carnosic acid dilutions was applied. MTT data showed that even the highest concentration of carnosic acid did not change the viability of the Vero cells (Figure 3C). qPCR data showed that there was a tendency for reduction in HSV-2 genome accumulation at the 3.1–1.6 μg/mL concentration range, but the first significant inhibitory concentration was 6.2 μg/mL (Figure 3D). The average HSV-2 genome reduction at the 6.2 μg/mL concentration was 10.95-fold; therefore, the carnosic acid IC_50_ value was between 3.1 and 6.2 μg/mL, and the IC_90_ value was approximately 6.2 μg/mL on the extracellular virions.

The same experimental setup was used to measure the carnosic acid antiviral activity using the human cervical epithelial cell line HeLa. Cytotoxicity measurement showed that the carnosic acid significantly reduced HeLa viability at the 100–50 μg/mL concentrations after 24 h (Figure 3E). The measurement of the carnosic acid activity on the intracellular HSV-2 showed that HSV-2 genome accumulation was significantly inhibited by the 100–25 μg/mL carnosic acid concentrations (Figure 3F). The 25 μg/mL carnosic acid concentration resulted in an average of 24.29-fold reduction in HSV-2 concentration; therefore, the carnosic acid IC_50_ and IC_90_ concentrations were between 25 and 12.5 μg/mL for the intracellular HSV-2. Similarly to Vero cells, the carnosic acid did not influence HeLa viability in the experimental setup used to test its antiviral activity on the extracellular virions (Figure 3G). qPCR data showed that there was a tendency for HSV-2 genome accumulation reduction at the 6.25–1.6 μg/mL concentration range, but the lowest significant inhibitory concentration was 12.5 μg/mL (Figure 3H). The average HSV-2 genome reduction at the 12.5 μg/mL concentration was 23.26-fold; therefore, the carnosic acid IC_50_ and IC_90_ values were smaller than 12.5 μg/mL on the extracellular virions.

### 2.4. Proteomics Analysis of Carnosic Acid Treated HeLa Cells

**Figure 4:**
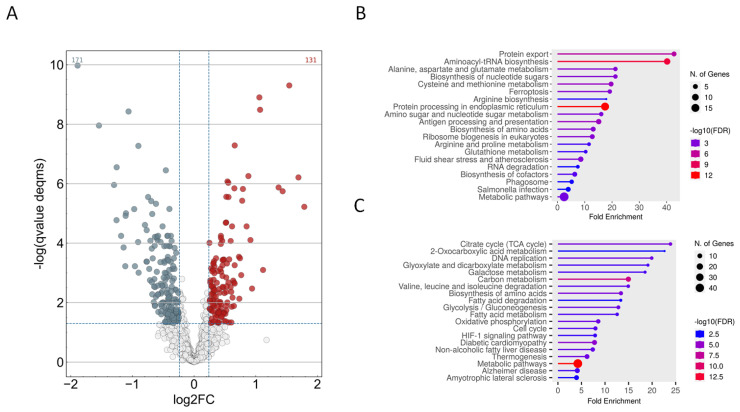
Proteomics analysis of carnosic acid treated HeLa cells. HeLa cells were treated with 25 μg/mL carnosic acid for 24 h. Five replicates were made and were compared to the untreated control cells. (**A**), Volcano plot of the upregulated and downregulated proteins in the carnosic acid-treated HeLa cells. (Carnosic acid/control fold change ≥1.18 with q-value < 0.05 (red color); carnosic acid/control fold change ≤1/1.18 with q-value < 0.05 (grey color)). (**B**), Functional analysis of the upregulated proteins. Graph shows the most significantly altered functional groups/pathways. (**C**), Functional analysis of the downregulated proteins. Graph shows the most significantly altered functional groups/pathways. Plot sizes for both the up- and downregulated proteins indicate the number of proteins related to a category, and plot color indicates the significance (−log_10_ FDR) of protein enrichment in a category.

**Figure 5:**
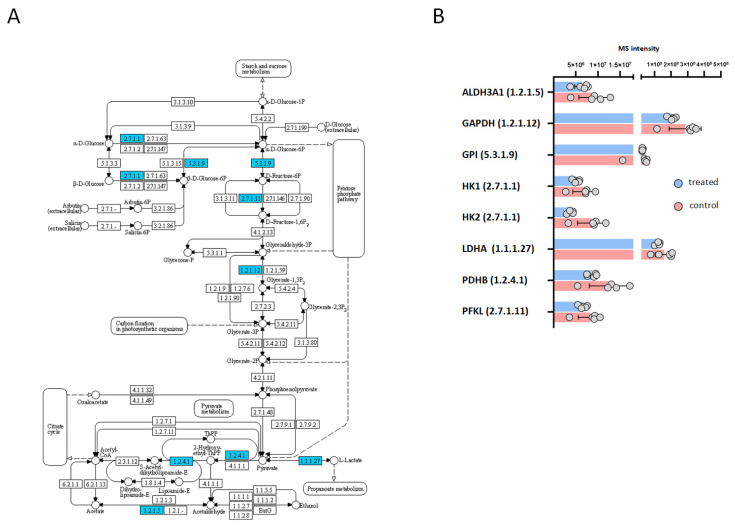
KEGG pathway analysis of carnosic acid downregulated proteins associated with glycolysis/gluconeogenesis. (**A**,**B**), Glycolysis/gluconeogenesis pathway and its downregulated proteins. The mapping of significantly downregulated proteins onto KEGG pathways was performed by ShinyGO software. Downregulated proteins of the pathway were labeled blue.

**Figure 6:**
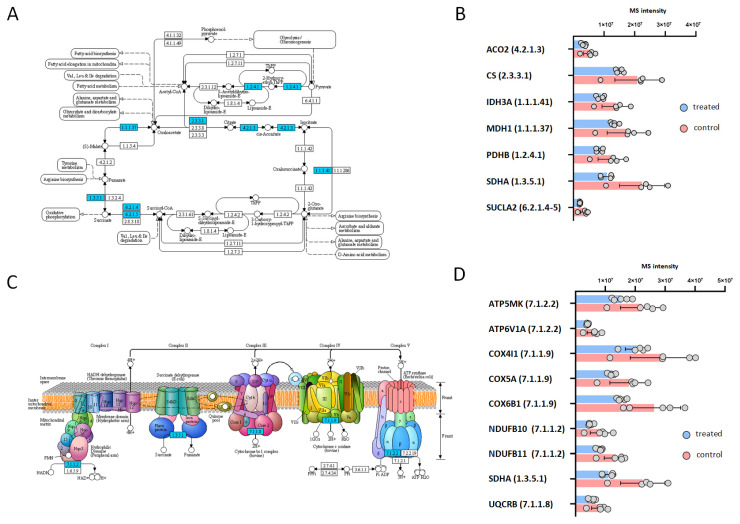
KEGG pathway analysis of carnosic acid downregulated proteins associated with citrate cycle and oxidative phosphorylation. (**A**,**B**), Citrate cycle (TCA cycle) pathway and its down-regulated proteins. (**C**,**D**), Oxidative phosphorylation pathway and its downregulated proteins. The mapping of significantly downregulated proteins onto KEGG pathways was performed by ShinyGO software. Downregulated proteins of the pathways were labeled blue.

In order to obtain insight into the possible antiviral actions of carnosic acid, we analyzed the protein expression of HeLa cells treated with 25 μg/mL carnosic acid for 24 h. At this time point, carnosic acid induced the expression of 121 proteins and repressed 171 proteins (Figure 4A). Functional analysis of the upregulated proteins showed that functional categories and pathways related to protein expression and folding were significantly enriched (Figure 4B). Functional categories such as biosynthesis of amino acids, aminoacyl-tRNA biosynthesis, protein processing in endoplasmic reticulum, and protein export were significantly enriched by the upregulated proteins. Interestingly, other categories related to inflammation and immunity such as “antigen processing and presentation” and “IL-17 signaling”, “NOD-like receptor signaling” were also significantly enriched. Many of the carnosic acid downregulated proteins were related to cellular metabolism (Figure 4C). Certain downregulated metabolic pathways were related to or involved in ATP generation, such as “glycolysis/gluconeogenesis”, “citrate cycle (TCA cycle)”, and “oxidative phosphorylation”. The Kyoto Encyclopedia of Genes and Genomes (KEGG) database search also showed that eight proteins in the “glycolysis/gluconeogenesis” pathway were downregulated (Figure 5A,B). The most highly downregulated (2.38-fold) protein was hexokinase-2 (HK2), a key enzyme that catalyzes the first step of aerobic glycolysis. The TCA cycle pathway contained seven downregulated proteins (Figure 6A,B), while the “oxidative phosphorylation” pathway contained nine downregulated proteins (Figure 6C,D). In both pathways, the most highly downregulated (1.87-fold) was the succinate dehydrogenase (SDHA), an enzyme that converts succinate to fumarate in the TCA cycle and also part of the complex II of the mitochondrial respiratory chain.

### 2.5. Mitochondrial Effects of Carnosic Acid

**Figure 7:**
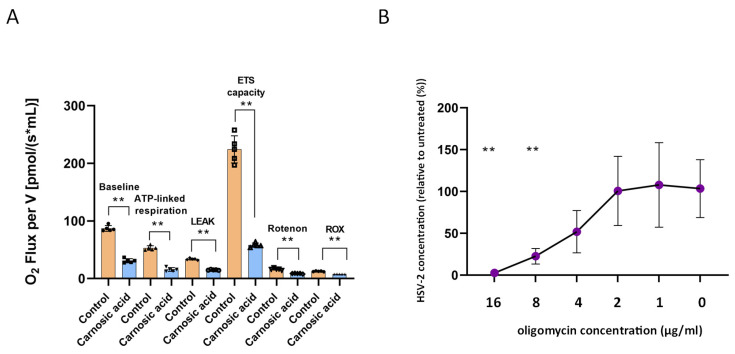
Mitochondrial effects of carnosic acid. (**A**), High-resolution respirometry of carnosic acid-treated HeLa cells. ROUTINE-, ATP-linked respiration, LEAK, and maximal capacity of the electron transport system (ETS) were significantly attenuated in carnosic acid-treated HeLa cells in comparison with control. The comparison of oxygen consumption values was performed by the Mann–Whitney U test ** *p* < 0.01. (**B**), Impact of inhibition of oxidative phosphorylation on HSV-2 replication. After HSV-2 infection, culture media were replaced with a media containing oligomycin at a 16–0 μg/mL concentration. HeLa cells were incubated for 24 h, 37 °C, 5%CO_2_. HSV-2 replication was measured by direct qPCR. Statistical analysis of qPCR data was performed by one-way ANOVA analysis corrected by Dunnett’s multiple comparison test. **: *p* < 0.001.

The impact of carnosic acid treatment on mitochondrial respiration was profound, as evidenced by the results presented in Figure 7A. A noticeable decrease was observed across various indicators following the administration of carnosic acid. The baseline respiration (ROUTINE) exhibited a significant difference between the carnosic acid-treated and untreated groups (87.08 ± 5.03 vs. 31.01 ± 3.66; *p* < 0.01). Similarly, LEAK respiration in the non-phosphorylating state was notably lower in the carnosic acid-treated cells compared to the control group (15.12 ± 0.64 vs. 33.84 ± 1.56; *p* < 0.01). Moreover, the maximal oxygen consumption achieved at an optimal uncoupler concentration, serving as an indicator for maximal electron transport system (ETS) capacity, was markedly reduced following carnosic acid treatment. The data revealed a nearly 4-fold decrease (*p* < 0.01) in this parameter in the treated cells (58.07 ± 3.81) compared to the controls (224.36 ± 23.49) after multi-step CCCP titration. Furthermore, mitochondrial respiration was significantly affected by rotenone, with a notable difference observed between the two groups (8.82 ± 0.61 vs. 16.52 ± 1.75; *p* < 0.01). Additionally, the ATP-linked respiration, obtained by subtracting LEAK respiration from the basic respiratory oxygen consumption values, indicated a substantial reduction in the phosphorylation-linked respiratory capacity in cells treated with carnosic acid compared to the untreated cells (15.89 ± 3.22 vs. 53.23 ± 3.88; *p* < 0.01). In conclusion, the findings suggest that carnosic acid treatment led to a significant reduction in the ATP synthesis capacity of the cells. These results underscore the impact of carnosic acid on mitochondrial respiration and highlight its potential implications for cellular function. To assess the role of mitochondrial respiration in HSV-2 replication in HeLa cells, the oxidative phosphorylation inhibitor oligomycin was added to the cell culture media after HSV-2 infection at a final concentration of 16–1 μg/mL for 24 h. qPCR data showed a significant, 5.39- and 39.94-fold reduction in HSV-2 genome accumulation by the 8 μg/mL and 16 μg/mL oligomycin concentrations, respectively (Figure 7B), indicating that intact oxidative phosphorylation is essential for HSV-2 replication in HeLa cells.

## 3. Discussion

Plants have most likely been used as medicine from the dawn of mankind [26]. The discovery of antibiotics temporarily made plant-based antimicrobials obsolete, but the emergence of resistant microorganisms made the research of natural antimicrobials attractive again. Here, we used the ethanol extracts of eight plants that grow in the southern part of Hungary.

We showed that—depending on the concentration—all plant extracts showed activity against HSV-2. Among these plants, *S. rosmarinus* decreased HSV-2 replication (genome accumulation) by approximately 90% at the lowest 0.47 mg/mL concentration. *S. rosmarinus* contains various already described antimicrobial compounds, such as rosmarinic acid, rosmaridiphenol, carnosol, epirosmanol, carnosic acid, rosmanol, and isorosmanol [27]. Carnosic acid is a major polyphenolic diterpene component of *S. rosmarinus*. As it was mentioned before, carnosic acid showed antiviral activity against various viruses; therefore, we chose this compound to test its antiviral action against HSV-2. We tested its antiviral activity against the extracellular form of HSV-2 by preincubating the virion before infection and also tested its activity against the intracellular form of HSV-2 by applying the carnosic acid after infection. Also, we used two different cell lines, Vero, an African green monkey kidney epithelial cell, which is a standard cell line used for HSV-2 propagation, and the human cervical epithelial cell line HeLa as a more relevant model of the human genital tract infections. Our results showed that in both cell lines, carnosic acid was capable of inhibiting HSV replication by inhibiting both the extracellular and intracellular forms of the virus. Altogether, the Vero cell line system showed higher sensitivity (lower inhibitory concentrations) to detect the antiviral actions of carnosic acid compared to the HeLa cells, which can be explained by the different host and tissue origins of these cell lines.

The fact that the carnosic acid could inhibit the intracellular form of HSV-2 directed us to explore the possible inhibitory mechanism(s). To obtain an unbiased view of the impact of carnosic acid on HeLa cells, we applied LC-MS/MS-based proteomics. This analysis showed that the carnosic acid had a significant impact on the HeLa proteome influencing the expression of proteins with a possible role in virus replication. Among the upregulated proteins, functional categories related to innate and adaptive immunity were identified, indicating that besides the direct influence on the HeLa epithelial cells, the carnosic acid may have a more general immune response modifier effect. Further studies are needed to clarify these potential effects of carnosic acid. Among the downregulated proteins, several were related to pathways of ATP generation such as glycolysis, TCA cycle, and terminal oxidation. Since viruses are not capable of macromolecule biosynthesis and energy production, they rely on intact host cell metabolism [28]. From this viewpoint, it is interesting that carnosic acid downregulated three basic metabolic pathways that were involved in host energy production. To test whether the proteome changes resulted in a depletion of energy production in HeLa cervical epithelial cells, we performed high-resolution respirometry. This analysis showed that carnosic acid suppressed four key mitochondrial respiration parameters such as (1) basal (or baseline), (2) ATP-respiration linked-, (3) maximal-, and (4) proton leak-linked oxygen consumption. Basal respiration is registered in the absence of inhibitors of ETS complexes, showing the energetic demand of cells under basal conditions. ATP-linked respiration is illustrated by the decrease in oxygen consumption after the addition of the ATP synthase inhibitor (oligomycin), which is the portion of basal respiration. The remaining basal respiration not coupled to ATP synthesis following oligomycin addition reflects the proton leak. Carnosic acid treatment markedly decreased all these three mitochondrial indices. Similarly, maximal respiration (or maximum ETS capacity measured by stepwise titration of the uncoupler CCCP) was attenuated by carnosic acid. Validating our proteomics and cellular respirometry data, we showed that inhibition of oxidative phosphorylation by oligomycin significantly suppressed HSV-2 replication. While there are conflicting results, HSV was shown to rely on host glycolysis, TCA cycle, and terminal oxidation for its replication. HSV-1 was shown to activate phosphofructo-1-kinase eventually and hexokinase, key enzymes of the glycolysis pathway in Vero cells, and inhibition of glycolysis resulted in significant inhibition of viral replication [29]. Other studies also showed that inhibition of glycolysis resulted in a significant decrease in HSV propagation [30,31]. There are differing results regarding the impact of HSV infection on the TCA cycle and oxidative phosphorylation including the potential shift from the TCA cycle and oxidative phosphorylation to the pentose phosphate pathway [32]. However, it was also shown previously that the HSV-1 UL43 protein was localized to the mitochondria, and increased aerobic oxidation and oxidative phosphorylation of glucose resulted in an overall increase in ATP generation [33]. The HSV-1 protein UL-16 also targets mitochondria and increases oxidative phosphorylation and ATP generation [34]. Moreover, inhibition of terminal oxidation was shown to inhibit HSV-1 replication in human umbilical cord-derived mesenchymal stem cells [35].

Altogether, our results showed that alcoholic extracts of medicinal plants are able to inhibit HSV-2 replication. The rosemary compound carnosic acid could inhibit both the extracellular and intracellular forms of the virus indicating that this compound or its derivatives may be used as antivirals against HSV. The antiviral effect of carnosic acid in the host may not be direct but acts via the host metabolism. Inhibition of ATP synthesis is one of the major effects of carnosic acid that could lead to significantly lower viral replication. Since viral replication is generally energy-dependent [36], carnosic acid may also potentially be used against other viruses.

## 4. Materials and Methods

### 4.1. HSV-2 Strain and Plant Extracts

An in-house isolated HSV-2 strain was grown in Vero cells and the infectious titer was measured in the same cells by using the plaque titration method [37]. The leaves of *M. officinalis*, *U. dioica*, *S. rosmarinus*, *A. officinalis*, *S. nigra*, *N. cataria*, *L. angustifolia*, and *O. vulgare* were collected in Békés county of Hungary and subsequently washed, air-dried and ground. A 50 g amount of plant material was used for extraction in 150 mL ethanol (Sigma, St. Louis, MO, USA).

### 4.2. 3-(4,5-dimethylthiazol-2-yl)-2,5-diphenyltetrazolium Bromide (MTT) Assay

MTT assay was carried out to identify the toxicity of the plant extracts and carnosic acid (Sigma, St. Louis, MO, USA). The minimum essential medium (MEM), 10% FBS medium on overnight grown Vero and HeLa cells was changed to media containing 2-fold dilutions of the tested compounds starting at 60 mg/mL of plant extracts and 100 μg/mL for carnosic acid and incubated 37 °C, 5% CO_2_. To test the short-term and long-term toxic effects of the compounds, the media was changed after 30 min and 24 h of incubation, respectively. MTT assay was performed 24 h post-treatment as described earlier [38]. Statistical analysis of MTT data was performed by comparison of the MTT values of untreated and treated samples by one-way ANOVA analysis corrected by Dunnett’s multiple comparison test.

### 4.3. Direct qPCR Assay of Antiviral Activity

Vero and HeLa cells (ATCC, Köln, Germany) were grown in 96-well plate with a density of 5 × 10^4^ cells/well in 100 µL MEM, 10% FBS. To test the antiviral activity of the plant extracts and carnosic acid on the extracellular HSV-2, the compounds were incubated with the HSV-2 virions for 30 min at 37 °C and the host cells were infected with the treated HSV-2, multiplicity of infection (MOI) of 0.1 for 60 min, 37 °C. After the incubation, the unbound virions were washed away with PBS, and fresh medium was added to the cells. To test the antiviral activity of the plant extracts and carnosic acid on the intracellular HSV-2, the host cells were infected with HSV-2 (MOI 0.1) for 60 min, 37 °C, and the medium was replaced with medium containing the tested compounds. At 24 h post-infection, the infected cells were washed with PBS and resuspended in 100 μL Milli-Q water (Merck, Burlington, MA, USA). The DNA extraction was performed by two freeze–thaw cycles of the cells. The direct qPCR was performed on the cell lysates in a Bio-Rad CFX96 real-time system as described earlier [25]. Briefly, 1 μL of the lysates was used as a template in a qPCR. The following HSV-2 specific primers were used: gD2-F: 5′-TCA GCG AGG ATA ACC TGG GA-3′, gD2-R: 5′-GGG AGA GCG TAC TTG CAG GA-3′. The qPCR reactions were performed using 2 µL HOT FIREPol EvaGreen supermix (Solis BioDyne, Tartu, Estonia), 1-1 µL of forward and reverse primers (10 pmol/µL each), 1 µL template and 5 μL MQ water. The qPCR protocol consisted of a 95 °C 12 min activation step followed by 45 cycles of 95 °C 15 s, 64 °C 30 s and 72 °C 30 s. The fluorescence intensity was detected at the end of the extension step. For each PCR, the cycle threshold (Ct) corresponding to the cycle where the amplification curve crossed the baseline was determined. For the relative DNA concentration calculation, the Ct data of the treated samples were compared to the untreated controls using the 2^ΔCt^ formula. Statistical analysis of qPCR data was performed by comparison of the Ct values of untreated and treated samples by one-way ANOVA analysis corrected by Dunnett’s multiple comparison test, similarly as described earlier [39].

### 4.4. Sample Preparation for Mass Spectrometry Analysis

Carnosic acid treated (25 μg/mL, 24 h) and untreated HeLa whole-cell lysates (*n* = 5) were produced by ultrasonic homogenization of 5 million cells in 500 µL buffer containing NaCl (150 mM), 2% sodium dodecyl sulfate, 1% sodium deoxycholate, and 2% IGEPAL CA-630. Prior to digestion, the protein contents of all samples were determined using BCA Protein Assay (Thermo Scientific, Rockford, IL, USA) according to the manufacturer’s acetone precipitation protocol. For all samples, 10 µg protein was processed by on-pellet digestion. Briefly, the samples were reduced with 10 mM dithiothreitol at 60 °C for 30 min and alkylated with 20 mM iodoacetamide in dark at room temperature for 30 min. The protein content was precipitated by adding a 7-fold volume of ice-cold acetone and incubated at −20 °C overnight. After centrifugation with 15,000× *g*, 10 min, 4 °C the supernatant was discarded. The protein pellet was washed twice with 500 µL acetone/water (85:15, *v*/*v*) mixture. After centrifugation with 14,000× *g*, 10 min, 4 °C, the protein pellet was dissolved in 15 µL RapiGest SF Surfactant (Waters, Milford, MA, USA) and was incubated at 100 °C for 5 min. After being cooled to room temperature, 65 µL ammonium bicarbonate buffer (AmBic, pH 8.0, 100 mM) and 0.25 µg trypsin in 5 µL AmBic were added to the mixtures. The samples were incubated at 37 °C for 30 min and another 0.25 µg trypsin in 5 µL AmBic was added, and the mixture was digested at 37 °C for 5.5 h. Digestion was stopped by the addition of 1 µL concentrated formic acid. After centrifugation, 5 µL of the supernatant was injected into the LC-MS/MS system.

### 4.5. LC-MS/MS Analysis

All measurements were carried out on a Waters ACQUITY UPLC M-Class LC system (Waters, Milford, MA, USA) coupled with an Orbitrap Exploris 240 mass spectrometer (Thermo Fisher Scientific, Waltham, MA, USA). Symmetry C18 (100 Å, 5 µm, 180 µm × 20 mm) trap column was used for trapping and desalting the samples. Chromatographic separation of peptides was accomplished on an ACQUITY UPLC M-Class Peptide BEH C18 analytical column (130 Å, 1.7 µm, 75 µm × 250 mm) at 45 °C by gradient elution. Water (solvent A) and acetonitrile (solvent B), both containing 0.1% formic acid were used as mobile phases at a flow rate of 200 nL/min. The sample temperature was maintained at 5 °C. The mass spectrometer was operated using the equipped Nanospray Flex Ion Source. Data were collected using the data-independent acquisition (DIA) method between 380 and 985 Th with precursor isolation windows of 10 Th width at 11,250 resolution to hit an AGC target 2 × 10^5^ and the maximum inject time was set to auto. Data acquisition was performed using Xcalibur^TM^ 4.6 (Thermo Fisher Scientific, Waltham, MA, USA). Raw LC-MS data files were processed using the DIA-NN 1.8.2 beta 22 [40]. For the analysis, a predicted spectral library was created using DIA-NN for the tryptic peptides of the Uniprot Human reference proteome assuming 2 missed cleavage sites and oxidation of methionine as variable modification (9,086,688 precursor ions of 20,575 proteins). Quantitative report of DIA-NN was processed using the MS-DAP R package [41]. The VSN normalization method was applied before differential expression analysis using the DeqMS algorithm. Precursor identifications were filtered at 1% FDR level in minimum of 50% of the samples of either treated or control group. For the differential expression analysis, a cut-off value of 0.05 was applied to the FDR-corrected *p*-values and a perturbation-based minimal fold change threshold was calculated for the actual dataset (log2 fold change threshold: 0.239). Functional classification of the significantly altered proteins was performed by the ShinyGO software version ShinyGO 0.80 [42]. LC-MS/MS data can be found in the Supplementary Materials.

### 4.6. Measurement of Mitochondrial Oxygen Consumption Using High-Resolution Respirometry

To measure mitochondrial respiration after 24 h of carnosic acid treatment, 3 × 10^6^ HeLa cells and untreated controls (*n* = 5) were suspended in MEM medium and gently pipetted into the chambers of the oxygraph (Oxygraph-2k; Oroboros Instruments, Innsbruck, Austria). All experiments were performed in 2 mL of respiration medium under continuous magnetic stirring (750 rpm) at 37 °C [43]. In order to prevent the effects of low O_2_ concentrations on mitochondrial respiration, the chamber O_2_ levels were maintained within the range of 50–200 μM without reoxygenation [44]. After reaching a stable baseline (ROUTINE) respiration ATP synthase (complex V) was inhibited with oligomycin (2.5 μM; LEAK respiration). The maximal capacity of the electron transport system (ETS) was achieved by stepwise titration of an uncoupler (carbonyl cyanide m-chlorophenylhydrazone [CCCP]; final concentration: 0.25 μM/step). Following complex I blockade with rotenone (0.5 μM), ETS-independent respiration (or residual oxygen consumption [ROX]) was assessed in the presence of the complex III inhibitor antimycin A (2.5 μM). DatLab 7.3 software (Oroboros Instruments) was utilized for online display respirometry data acquisition and analysis.

## Data Availability

Data supporting the reported results can be found in the Supplementary Materials.

## Supplementary Materials

The following supporting information can be downloaded at: https://www.mdpi.com/article/10.3390/ijms25094983/s1.
